# Association of rs4680 *COMT*, rs6280 *DRD3*, and rs7322347 *5HT2A* With Clinical Features of Youth-Onset Schizophrenia

**DOI:** 10.3389/fpsyt.2019.00830

**Published:** 2019-11-12

**Authors:** Anna Morozova, Yana Zorkina, Konstantin Pavlov, Olga Pavlova, Zinaida Storozheva, Eugene Zubkov, Natalia Zakharova, Olga Karpenko, Alexander Reznik, Vladimir Chekhonin, Georgiy Kostyuk

**Affiliations:** ^1^Department Basic and Applied Neurobiology, V. Serbsky Federal Medical Research Centre of Psychiatry and Narcology, Moscow, Russia; ^2^N.A. Alekseev Psychiatric Clinical Hospital № 1, Moscow, Russia; ^3^Department of Medical Nanobiotechnology, Pirogov Russian National Research Medical University, Moscow, Russia

**Keywords:** single-nucleotide polymorphism, schizophrenia, youth-onset, 5-hydroxytryptamine 2A receptors, dopamine receptor D_3_, catechol-O-methyltransferase

## Abstract

We investigated the associations of rs4680 *COMT*, rs6280 *DRD3*, and rs7322347 *5HT2A* with youth-onset schizophrenia in the Russian population in a case–control study, and the role of the genotype in the severity of clinical features. The association between rs7322347 and schizophrenia (p = 0.0001) is described for the first time. Furthermore, we found a link with rs6280 and rs4680 in females (p = 0.001 and p = 0.02 respectively) and with rs7322347 in males (p = 0.002). Clinical symptoms were assessed on three scales: the Clinician-Rated Dimensions of Psychosis Symptom Severity scale, Positive and Negative Syndrome Scale, and Frontal Assessment Battery. Gender differences in clinical features are of particular interest. In our study we found gender differences in the severity of clinical features—higher scores for delusions (Positive and Negative Syndrome Scale and Diagnostic and Statistical Manual of Mental Disorders, Fifth Edition) in males and higher scores for depression, delusions, somatic concern, motor retardation, poor attention were found in females.

## Introduction

Schizophrenia, as a chronic progressive disease, is a socially significant problem, primarily due to the fact that the unemployment rate among people diagnosed with schizophrenia reaches 80–90% (1). Most often, schizophrenia affects young people of working age. According to statistics, the prevalence of the disease ranges from 0.8–1% of the population (2). In 40% of cases, a relapse of psychotic symptoms is observed within a year after the initial hospitalization. In 40–80% of patients, the course of the disease becomes chronic (3). The life expectancy of schizophrenic patients is on average 15–20 years shorter than in the general population (4). A genome-wide association study has established independent associations between schizophrenia and single-nucleotide polymorphisms (SNPs) in 145 loci called “schizophrenia risk loci” (5). However, none of them can be considered as an absolute predictor of schizophrenia.

In addition, with respect to the majority of genes associated with schizophrenia, one cannot confidently say that a particular gene is responsible for a specific phenotypic trait. Candidate genes may occur in only 2–5% of patients, and the odds ratio rarely reaches 1:20. There is also no evidence of a stable association of genomic variations with forms of schizophrenia (6). Data on the association of certain genetic polymorphisms with particular manifestations of schizophrenia are scarce. Examples are links: between polymorphism in solute carrier family 6 member 4 (*SLC6A4*) gene with defect in facial emotion recognition in schizophrenia (7), between polymorphism in 2’3’-cyclonucleotide 3’-phosphodiesterase (*CNP*) gene with psychopathological symptoms including catatonia, depression, anxiety, and autism manifestations (8) and polymorphism in a brain-derived neurotrophic factor (*BDNF*) gene with symptoms of insertion and withdrawal of thoughts, verbal hallucinations, and delusions (9).

Selection of a homogeneous cohort of patients in studies of genetic associations with phenotypic manifestations is a key factor in this type of research. The age of onset has been considered the important characteristic of schizophrenia that may yield clues to its origin, and there is strong evidence for an underlying genetic contribution to the age of onset in schizophrenia (10). Comparatively there are not enough studies to understand clearly how genetic risk factors influence the development of youth-onset schizophrenia, especially gender differences in expression of the disease.

Epidemiological studies exploring geographical variation in the incidence of psychotic disorders have highlighted various characteristics of environments that are typically associated with an increased risk, including high population density, urbanicity, income deprivation, income inequality and low social cohesion.

The empirical findings on the association between income inequality and mental health are not clear cut. The extant literature in the field is characterised by small effect sizes and a high level of heterogeneity in findings (11). Socioeconomic status alone does not increase the likelihood of youth-onset schizophrenia but it somehow magnifies the deleterious effect of early onset schizophrenia on negative symptoms (12).

In our opinion genetic factors play a larger role in the development of schizophrenia with youth-onset. We consider that neuropathology, neurodegenerative and environmental factors are more important than genetic factors for the development of schizophrenia in later-onset groups of patients (13–15). Stress produces altered neural processing and durability of gray-matter changes (16, 17). According to the literature, age at the onset of schizophrenia is defined as the age when positive symptoms first become apparent (18). Youth-onset schizophrenia presents most commonly during early adulthood (late teens to age 26) (19). Middle-onset (between 26 and 40 years) and late-onset schizophrenia (after 40 years of age) involve a different interplay of risk factors compared with the youth-onset form of the disease (18). The association of some SNPs with age of onset of the disease has been shown, as well as the association with symptoms resistant to therapy (20, 21). Most studies find that the age of onset for women is several years later than for men (22).

There are insufficient genetic studies of youth-onset schizophrenia in ethnically and socio-economic homogenous samples. We decide to use in our study a homogenous groups of men and women with positive symptoms of schizophrenia that first become apparent in the age period 18 to 26.

### Single-Nucleotide Polymorphisms Selection

Catechol-*O*-methyltransferase (COMT) is an enzyme that is involved in the metabolism of catecholamines (epinephrine, norepinephrine, and dopamine). The *COMT* gene is located in the chromosomal region 22q11.1–q11.2. It contains a single-nucleotide substitution of G472A in exon 4, which results in the replacement of the amino acid valine with methionine (Val158Met polymorphism). This replacement (Met allele) leads to a decrease in enzyme activity (23) that can influence the catecholamines turnover in the prefrontal cortex. *COMT* Val158Met is widely regarded as potentially important for understanding the genetic etiology of many different psychiatric disorders, and the results of numerous studies suggest pleiotropy and the importance of examining subgroups defined by variables such as age of onset, sex, and ethnicity (24).

The dopamine receptor D_3_ (DRD3) is a D_2_-like receptor and is expressed in the ventral striatum. The *DRD3* gene is located on chromosome 3 (3q13.31) and has 12 exons and five introns. The rs6280 polymorphic locus is a C/T single-nucleotide substitution that leads to the replacement of serine with a glycine residue at position 113890815 of the N-terminal domain of the extracellular receptor (Ser9Gly) (25). The *DRD3* gene is considered a candidate gene for schizophrenia: in patients with this disease, an increase in the density of DRD3 in the striatum region has been found, together with a relative accumulation of “truncated forms” of receptor protein formed during abnormal splicing. DRD3 receptors are involved in the mechanisms of affective reactions and cognitive processes (26).

The 5-hydroxytryptamine *2A* receptors (5HT2A) are class A heptahelical receptors activated by serotonin (5-hydroxytryptamine, 5-HT) that signal, at least in part, by activating heterotrimeric G proteins of the Gα_q-11_ subtype (27). The 5-HT2A receptors are expressed at high levels on pyramidal cells of the prefrontal cortex, where they are ideally positioned to modulate both cognitive functions, such as working memory or executive control, and also emotions, through dynamic interactions with the amygdala (28). Serotonin receptors are also distributed along the midbrain periaqueductal gray (PAG) and the hypothalamus (29). The 5-HT2A receptors, especially those located in the prefrontal cortex, play an important role in the pathophysiology and therapeutics of neuropsychiatric disorders, including schizophrenia and depression (30–33).

During the last decade, several groups have investigated SNPs of the *HTR2A* gene in connection with psychiatric and personality disorders (34–39). Most of the observations have been made with regard to the number of variations located in the promoter or the coding region; however, literature data also indicate that intronic variant rs7322347 might well be of interest with regard to behavioural aspects, as it has shown marked association with the combined subtype of childhood attention-deficit hyperactivity disorder and with suicide attempts in females subjected to physical assault at a younger age (40, 41). In a recent study, a robust contribution of the rs7322347 variation within the gene encoding serotonin receptor 2A to aggressive traits, was demonstrated (42).

We decided to investigate the association of rs4680 *COMT*, rs6280 *DRD3*, and rs7322347 *5HT2A* with youth-onset schizophrenia in a homogenous Russian population, because of their prominence in the literature. Associations between schizophrenia and SNPs in *COMT* and *DRD3* genes are still controversial, there are studies both confirming and refuting these relationships (43, 46). SNP in *5HT2A* gene associated with aggression and suicidal behavior (42, 41), so we have a hypothesis about the link between this SNP and schizophrenia.

Due to genetic heterogeneity between different populations, it is necessary to conduct separate studies for each one and results obtained in one population may not apply elsewhere.

The aim of our study is to find which of these SNPs is most strongly associated with our sample of youth onset schizophrenia patients. In the future, we would like to find a panel of objective markers that would help doctors predict the type and character of pathology and make decisions accordingly.

## Materials and Methods

### Participants

The protocol of this study was approved by the Interdisciplinary Ethics Committee, Moscow (22/07/2017).

A total of 462 (186 male, 276 female, mean age 32.6 ± 11.6) healthy volunteer controls and 212 (100 male, 112 female, mean age 29 ± 8.4) patients who gave their consent to participating and met the inclusion criteria of youth-onset schizophrenia (from 18 to 26 years old), participated in the study. All participants (patients and control) were Slavs from the central part of Russia. This was clarified using questions about the nationality of the participants, their parents and grandparents. Patients were recruited from N. A. Alekseev Psychiatric Hospital No. 1, Moscow, Russia. Inclusion criteria were inpatients, diagnosed with schizophrenia. Next, we selected the patients with the onset of prodromal events prior to age 26 years old and after age 18. After inclusion, all patients underwent complete diagnostic evaluation. Exclusion criteria were intellectual disabilities, substance abuse and dependence and any comorbid severe somatic or neurological disorder. Patients were diagnosed by at least two psychiatrists according to the criteria of the Diagnostic and Statistical Manual of Mental Disorders (Fifth Edition, DSM-V). (47, 48). Patients were evaluated using a structured interview for the Positive and Negative Syndrome Scale (PANSS), including the PANSS positive, PANSS negative, and PANSS general psychopathology subscales (49). The Russian version of PANSS, with acceptable validity and reliability, was used to evaluate the severity of psychosis symptoms of schizophrenia patients in this study (50). The mean sum of PANSS scores was 104.15 ± 30.67. Cognitive conditions were examined using the Frontal Assessment Battery (FAB) (51).

Healthy controls matched with the patients in sex, age, and birthplace at the same time were randomly recruited from the volunteers who came to give a blood donation (Blood Center named after O.K. Gavrilov—a medical institution specializing in transfusion of blood and its components). Subjects were excluded due to any of the following: positive family histories (first-degree relatives) of psychiatric illness; substance abuse; abnormal birth; febrile convulsions; juvenile adoption or residence with a single-parent family; pregnancy or lactation, in addition, the socio-economic status of the participants was approximately the same, all participants were either university student or had completed university and came from the middle income families.

#### Genotyping

Genomic DNA samples were obtained from peripheral blood lymphocytes using an automatic DNA extraction (QIAGEN QIAcube) system, according to the manufacturer’s recommendations. DNA concentration and sample quality were assessed spectrophotometrically (NanoVue, GE Healthcare). The obtained DNA samples were normalized in TE buffer to a final concentration of 4 ng/µl in the 384-well plate format. All SNPs were typed using predesigned TaqMan SNP genotyping assays (Applied Biosystems, Thermo Fisher, USA). PCR and allelic discrimination assays were run using a QuantStudio 5 Real-Time PCR system (Applied Biosystems, Thermo Fisher, USA).

#### Statistical Analysis

Statistical assessment of the reliability of differences in the frequency distribution of polymorphic alleles and genotypes between a group of patients with schizophrenia and a group of healthy individuals, and the identification of genotype associations with individual symptoms was performed using the standard χ2 method. The strength of associations was evaluated in terms of the odds ratio and its 95% confidence interval (95% CI).

We assessed the associations between the SNP genotype and scales performances using one-way ANOVA in Statistica 7.0. Bonferroni corrections were applied to correct the p-values of alleles (corrected α = 0.05/3 = 0.017), to control inflation of the type I error rate.

All of the scales (PANSS, Clinician-Rated Dimensions of Psychosis Symptom Severity, and FAB) were two-tailed, with statistical significances of p < 0.05 using a *post hoc* Fisher analysis.

## Results

There were no significant differences between case and control groups in terms of gender, age, and years of education (p > 0.05). Genotype frequencies in all groups followed the Hardy–Weinberg equilibrium. Allele and genotype frequencies in the groups, as well as p-values of HWE, are shown in **Table 1**. Analysis revealed significant gender-specific associations between studied polymorphisms and youth-onset schizophrenia. Gender stratification showed that association with schizophrenia risk was significant for rs6280 and rs4680 in female subjects and for rs7322347 in male subjects (**Table 2**). That’s mean We found significant differences in clinical features between males and females on the Clinician-Rated Dimensions of Psychosis Symptom Severity and PANSS scales. The gender differences in the clinical characteristics of the patients are presented in **Table 3**. Analysis of the association between genotype and schizophrenia symptoms has been provided for male and female groups separately. The clinical characteristics of the patients across genotypes are presented in **Table 4**.

**Table 1:** Genotype frequencies of rs6280 (*DRD3*), rs4680 (*COMT*), and rs7322347 (*5HT2A*) in the control and youth-onset schizophrenia groups.

Gene	Genotype	Control				Case			
		Frequence	HWE	χ^2^	p	Frequence	HWE	χ^2^	p
DRD3	C/C	0,037	0,051	2,43	0,12	0,097	0,079	2,12	0,15
C/T	0,38	0,351	0,369	0,404					
T/T	0,584	0,598	0,534	0,516					
Male	C/C	0,043	0,054	1,05	0,31	0,057	0,059	0,02	0,88
C/T	0,378	0,357	0,374	0,369					
T/T	0,579	0,589	0,569	0,572					
Female	C/C	0	0,038	3,15	0,08	0,128	0,097	3,38	0,07
C/T	0,389	0,313	0,365	0,428					
T/T	0,611	0,649	0,506	0,475					
COMT	A/A	0,279	0,281	0,03	0,86	0,277	0,257	1,62	0,2
A/G	0,503	0,498	0,461	0,5					
G/G	0,218	0,221	0,262	0,243					
Male	A/A	0,23	0,245	1,03	0,31	0,305	0,263	3,37	0,07
A/G	0,53	0,5	0,415	0,5					
G/G	0,24	0,255	0,28	0,237					
Female	A/A	0,456	0,433	0,79	0,38	0,255	0,253	0,01	0,94
A/G	0,405	0,45	0,497	0,5					
G/G	0,139	0,117	0,248	0,247					
5HT2A	A/A	0,084	0,098	1,59	0,21	0,183	0,176	0,24	0,62
A/T	0,459	0,431	0,473	0,487					
T/T	0,457	0,471	0,344	0,337					
Male	A/A	0,07	0,095	4,25	0,04	0,152	0,176	1,25	0,26
A/T	0,477	0,426	0,536	0,487					
T/T	0,454	0,479	0,312	0,336					
Female	A/A	0,164	0,119	2,11	0,15	0,209	0,175	2,88	0,09
A/T	0,364	0,452	0,419	0,487					
T/T	0,473	0,428	0,372	0,338					

**Table 2:** Distribution of *DRD3*, *COMT*, and *5HT2A* polymorphisms in youth-onset schizophrenia and control groups.

rs	Allele	Case frequence	Control frequence	χ^2^	p	Odds ratio	(95% Cl)
rs6280 DRD3	C	0,281	0,227	4,97	0,03	1,34	1.04–1.72
Chromosome 3	T	0,719	0,773	0,75	0.58–0.97		
Male	C	0,244	0,232	0,13	0,72	1,07	0.75–1.51
T	0,756	0,768	0,94	0.66–1.33			
Female	C	0,311	0,194	5,39	0,02	1,87	1.10–3.19
T	0,689	0,806	0,54	0.31–0.91			
rs4680 COMT	A	0,507	0,53	0,66	0,42	0,91	0.73–1.14
Chromosome 22	G	0,493	0,47	1,1	0.88–1.37		
Male	A	0,513	0,495	0,22	0,64	1,07	0.79–1.46
G	0,487	0,505	0,93	0.69–1.26			
Female	A	0,503	0,658	10,14	0,001	0,53	0.35–0.78
G	0,497	0,342	1,9	1.28–2.83			
rs7322347 5HT2A	A	0,419	0,314	15,01	0,0001	1,58	1.25–1.99
Chromosome 13	T	0,581	0,686	0,63	0.50–0.80		
Male	A	0,42	0,308	9,88	0,002	1,63	1.20–2.21
T	0,58	0,692	0,61	0.45–0.83			
Female	A	0,419	0,345	1,8	0,18	1,37	0.87–2.15
T	0,581	0,655	0,73	0.46–1.15			

**Table 3:** The gender differences of clinical characteristics of the patients.

Scale		F	p	Male (M ± SD)	Female (M ± SD)
Clinician-Rated Dimensions of Psychosis Symptom Severity	Hallucinations	0,729	0,394	1,7 ± 1,57	1,89 ± 1,58
	Delusions	3,952	0,048	2,69 ± 1,3	2,33 ± 1,35
	Disorganized speech	0,904	0,343	1,96 ± 1,25	2,12 ± 1,18
	Abnormal psychomotor behavior	0,363	0,548	1,93 ± 1,27	1,82 ± 1,36
	Negative symptoms (restricted emotional expression avolition)	2,840	0,093	2,35 ± 1,18	2,61 ± 1,07
	Impaired cognition	0,323	0,571	2,01 ± 1,14	2,1 ± 1,14
	Depression	5,049	0,026	0,47 ± 0,97	0,79 ± 1,08
	Mania	0,001	0,975	0,41 ± 1,02	0,40 ± 1,04
PANSS	PI. Dellusions	4,548	0,034	4,71 ± 1,67	4,22 ± 1,69
	P2.Conceptual disorganization	3,989	0,047	4,04 ± 1,59	4,48 ± 1,59
	P3. Hallucinations	0,212	0,645	3,35 ± 2,23	3,49 ± 2,07
	P4. Excitement	9,096	0,003	3,49 ± 1,77	2,77 ± 1,68
	P5. Grandiosity	0,327	0,568	2,42 ± 1,77	2,29 ± 1,57
	P6. Suspiciousness/persecution	1,543	0,216	4,13 ± 1,73	3,85 ± 1,59
	P7. Hostility	3,591	0,059	3,2 ± 1,84	2,74 ± 1,7
	N1. Blunted affect	2,816	0,095	4,15 ± 1,76	4,53 ± 1,54
	N2. Emotional withdrawal	0,742	0,390	4,16 ± 1,83	4,37 ± 1,67
	N3. Poor rapport	3,028	0,083	3,68 ± 1,93	4,13 ± 1,81
	N4. Passive/apathetic social withdrawal	1,782	0,183	3,89 ± 1,83	4,22 ± 1,72
	N5. Difficulty in abstract thinking	3,258	0,073	3,98 ± 1,84	4,41 ± 1,58
	N6. Lack of spontaneity and flow of conversation	1,446	0,231	3,84 ± 1,85	4,14 ± 1,71
	N7. Stereotyped thinking	2,273	0,133	3,53 ± 1,77	3,89 ± 1,72
	G1. Somatic concern	5,065	0,025	1,83 ± 1,48	2,3 ± 1,54
	G2. Anxiety	0,643	0,424	3,14 ± 1,7	3,32 ± 1,61
	G3. Guilt feelings	2,085	0,150	1,69 ± 1,22	1,95 ± 1,42
	G4. Tension	0,237	0,627	3,77 ± 1,77	3,66 ± 1,59
	G5. Mannerisms and posturing	6,156	0,014	2,1 ± 1,4	2,61 ± 1,58
	G6. Depression	10,692	0,001	1,78 ± 1,35	2,46 ± 1,63
	G7. Motor retardation	5,656	0,018	2,13 ± 1,52	2,63 ± 1,54
	G8. Uncooperativeness	6,032	0,015	3,06 ± 1,81	3,65 ± 1,7
	G9.Unusual thought content	0,648	0,422	3,86 ± 1,68	4,05 ± 1,82
	G10. Disorientation	2,604	0,108	1,85 ± 1,38	2,19 ± 1,64
	G11. Poor attention	6,422	0,012	3,58 ± 1,49	4,13 ± 1,62
	G12. Lack of judgment and insight	0,728	0,394	4,37 ± 1,5	4,56 ± 1,62
	G13. Disturbance of volition	3,439	0,065	4,05 ± 1,85	4,49 ± 1,58
	G14. Poor impulse control	0,045	0,832	3,3 ± 1,88	3,24 ± 1,98
	G15. Preoccupation	3,111	0,079	3,61 ± 1,71	4,03 ± 1,72
	G16. Active social avoidance	1,327	0,251	3,93 ± 1,75	4,2 ± 1,63
	Total score	2,333	0,128	100,35 ± 31,97	106,86 ± 29,89
FAB	1. Similarities (conceptualization)	0,778	0,379	2,07 ± 0,87	1,96 ± 0,87
	2. Lexical fluency (mental flexibility)	0,470	0,494	2,02 ± 0,87	1,93 ± 0,87
	3. Motor series (programming)	0,352	0,554	2,10 ± 0,92	2,01 ± 0,96
	4.Conflicting instructions (sensitivity to interference)	0,582	0,447	2,16 ± 0,83	2,06 ± 0,9
	5. Go–No Go (inhibitory control)	0,075	0,784	1,65 ± 0,94	1,69 ± 0,89
	6.Prehension behavior (environmental autonomy)	1,276	0,260	2,35 ± 1,01	2,42 ± 0,96

**Table 4:** The clinical characteristics of the patients across genotypes.

Scale		DRD3 (Male)		DRD3 (Female)		COMT (Male)		COMT (Female)		5HT2A (Male)		5HT2A (Female)	
		F	p	F	p	F	p	F	p	F	p	F	p
Clinician-Rated Dimensions of Psychosis Symptom Severity	Hallucinations	1,38	0,24	0,20	0,66	0,13	0,72	1,47	0,23	0,80	0,37	0,07	0,80
	Delusions	1,86	0,18	0,50	0,48	1,31	0,26	0,03	0,87	**10,08**	0,002*	0,13	0,72
	Disorganized speech	1,80	0,18	0,80	0,37	0,38	0,54	3,83	0,05	0,02	0,90	0,01	0,94
	Abnormal psychomotor behavior	1,57	0,21	3,33	0,07	0,22	0,64	0,17	0,68	0,20	0,65	0,84	0,36
	Negative symptoms (restricted emotional expression avolition)	0,66	0,42	0,20	0,66	0,18	0,67	2,74	0,10	0,04	0,83	0,37	0,55
	Impaired cognition	0,67	0,42	0,44	0,51	0,86	0,36	1,65	0,20	0,00	0,97	0,55	0,46
	Depression	1,85	0,18	2,82	0,10	0,00	0,98	0,23	0,63	0,22	0,64	0,37	0,55
	Mania	0,96	0,33	0,69	0,41	0,02	0,90	0,44	0,51	0,23	0,63	0,00	0,98
PANSS	PI. Dellusions	**5,64**	**0,020**	0,36	0,55	1,79	0,18	3,32	0,07	**5,23**	**0,024**	0,03	0,86
	P2.Conceptual disorganization	0,84	0,36	1,93	0,17	0,54	0,46	2,41	0,12	0,97	0,33	0,12	0,73
	P3. Hallucinations	1,47	0,23	0,18	0,67	0,16	0,69	2,68	0,10	0,01	0,91	0,21	0,65
	P4. Excitement	1,38	0,24	1,79	0,18	1,56	0,21	0,43	0,52	0,91	0,34	0,67	0,41
	P5. Grandiosity	3,18	0,08	0,33	0,57	0,33	0,57	3,40	0,07	0,22	0,64	2,61	0,11
	P6. Suspiciousness/persecution	2,70	0,10	3,03	0,08	2,00	0,16	**5,56**	**0,020**	0,64	0,42	1,12	0,29
	P7. Hostility	**8,32**	0,005*	1,06	0,30	2,61	0,11	1,31	0,26	0,83	0,37	**6,95**	0,009*
	N1. Blunted affect	**4,95**	**0,03**	0,00	0,99	0,01	0,94	2,44	0,12	0,00	0,99	0,24	0,63
	N2. Emotional withdrawal	2,82	0,10	0,07	0,79	0,08	0,78	1,59	0,21	0,07	0,79	0,29	0,59
	N3. Poor rapport	3,14	0,08	1,82	0,18	0,40	0,53	**5,31**	**0,023**	1,27	0,26	0,46	0,50
	N4. Passive/apathetic social withdrawal	**4,85**	**0,03**	0,01	0,92	0,07	0,79	1,37	0,24	1,42	0,24	0,04	0,84
	N5. Difficulty in abstract thinking	**11,17**	0,001*	0,00	0,99	0,57	0,45	2,83	0,10	4,08	0,05	0,08	0,78
	N6. Lack of spontaneity and flow of conversation	3,49	0,06	1,53	0,22	0,00	0,99	1,77	0,19	0,07	0,79	0,49	0,48
	N7. Stereotyped thinking	2,30	0,13	0,04	0,83	0,78	0,38	3,15	0,08	0,18	0,68	0,01	0,91
	G1. Somatic concern	0,10	0,76	0,05	0,83	0,04	0,84	0,81	0,37	0,26	0,61	0,00	0,99
	G2. Anxiety	2,86	0,09	0,33	0,57	1,16	0,29	1,67	0,20	0,10	0,75	2,06	0,15
	G3. Guilt feelings	0,00	0,99	0,19	0,67	0,28	0,60	3,09	0,08	0,02	0,88	0,13	0,72
	G4. Tension	**6,65**	0,011*	0,18	0,67	0,38	0,54	3,86	0,05	2,58	0,11	0,00	0,97
	G5. Mannerisms and posturing	4,07	0,05	1,09	0,30	0,10	0,76	1,40	0,24	0,02	0,90	2,73	0,10
	G6. Depression	0,50	0,48	**5,27**	**0,024**	0,77	0,38	0,32	0,57	0,05	0,82	0,00	0,95
	G7. Motor retardation	0,05	0,83	0,06	0,81	0,05	0,83	0,63	0,43	**5,62**	**0,020**	0,01	0,92
	G8. Uncooperativeness	2,20	0,14	0,43	0,51	1,37	0,25	**6,15**	0,015*	0,00	0,98	0,05	0,82
	G9.Unusual thought content	1,67	0,20	0,20	0,65	0,04	0,84	2,83	0,10	0,47	0,49	0,31	0,58
	G10. Disorientation	0,13	0,72	2,78	0,10	0,72	0,40	1,89	0,17	1,13	0,29	3,43	0,07
	G11. Poor attention	3,48	0,07	0,04	0,84	0,69	0,41	**6,64**	0,011*	0,13	0,72	0,00	0,95
	G12. Lack of judgment and insight	2,26	0,14	0,11	0,74	0,07	0,79	0,48	0,49	0,66	0,42	0,01	0,93
	G13. Disturbance of volition	2,99	0,09	0,27	0,61	0,07	0,79	2,94	0,09	0,26	0,61	0,13	0,72
	G14. Poor impulse control	0,66	0,42	3,94	0,05	0,39	0,53	0,04	0,85	1,09	0,30	1,24	0,27
	G15. Preoccupation	1,41	0,24	0,04	0,85	0,56	0,46	2,28	0,13	0,17	0,68	0,02	0,88
	G16. Active social avoidance	2,86	0,09	0,05	0,82	0,05	0,82	3,86	0,05	0,11	0,75	0,11	0,74
	Total score	**5,82**	**0,018**	0,70	0,40	0,19	0,66	**6,30**	0,013*	0,01	0,93	0,23	0,64
FAB	1. Similarities (conceptualization)	0,13	0,72	0,08	0,78	0,21	0,65	**7,92**	0,006*	0,40	0,53	0,10	0,75
	2. Lexical fluency (mental flexibility)	0,27	0,60	0,05	0,83	2,31	0,13	**5,26**	**0,024**	0,21	0,65	0,00	0,96
	3. Motor series (programming)	0,07	0,80	0,68	0,41	0,41	0,52	**4,96**	**0,029**	0,01	0,93	0,03	0,86
	4. Conflicting instructions (sensitivity to interference)	0,01	0,93	0,19	0,66	0,60	0,44	**4,44**	**0,038**	0,12	0,73	0,47	0,50
	5. Go–No Go (inhibitory control)	1,63	0,21	0,54	0,47	0,08	0,78	**9,64**	0,002*	1,23	0,27	0,28	0,60
	6. Prehension behavior (environmental autonomy)	0,44	0,51	0,14	0,71	1,17	0,28	3,39	0,069	1,66	0,20	0,21	0,65

### Diagnostic and Statistical Manual of Mental Disorders (Fifth Edition; Clinician-Rated Dimensions of Psychosis Symptom Severity) for Rs7322347 Genotypes in Youth-Onset Schizophrenia Patients

In male patients (**Table 4**) ANOVA revealed a significant effect (F = 10.09; p = 0.002) of the *5HT2A* genotype on the delusions score. After Bonferroni correction, differences still remained in delusion scores: homozygotes for the minor allele A had significantly lower scores than men who were homozygotes or heterozygotes for the T allele (2.86 ± 1.21 and 1.71 ± 1.44).

### PANSS for Rs7322347 Genotypes in Youth-Onset Schizophrenia Patients

In men, differences were found on the PANSS scale for P1 (F = 5.23; p = 0.024) and G7 (F = 5.62; p = 0.02). In females, differences were found in the symptom P1 “hostility” (F = 6.95; p = 0.01). The association remained statistically reliable after Bonferroni correction. The presence of the homozygous genotype for the minor A allele is statistically significantly associated with an increase in the number of points for hostility in P7 (2.5 ± 1.64 and 3.6 ± 1.79).

### PANSS for Rs4680 Genotypes in Youth-Onset Schizophrenia Patients

Significant differences were found in the *COMT* rs4680 genotypes with regard to PANSS scores. Groups were identified which were homozygous for allele A with the allele G (homozygous A/G and heterozygous G/G). In men, no genotype associations were found with signs, according to the three scales studied. In women, differences were found on the PANSS scale for P6 (F = 5.64; p = 0.02), N5 (1.17 ± 1.03; 4.14 ± 1.79), G8 (F = 6.65; p = 0.014), G11 (F = 6.65; p = 0.014) and the overall PANSS score (F = 5.82; p = 0.018). It should be noted that for this polymorphism, associations of the genotypes with schizophrenia in case–control studies were also found in women only, but not in the total sample. Therefore, after correction for multiple comparisons, the differences remained significant in terms of G8, G11 and the overall PANSS score. Thus, the presence of the G allele in a homozygote or heterozygote increases the severity of the G8 traits (3.07 ± 1.8; 3.84 ± 1.63), G11 traits (3.51 ± 1.42; 4.32 ± 1.64) and the total PANSS score (97 ± 30.13; 109.88 ± 29.34).

### PANSS for Rs6280 Genotypes in Youth-Onset Schizophrenia Patients

Groups were identified which were homozygotes in the minor allele C with the allele T (homozygote T/T and heterozygote C/T). In men, differences were found on the PANSS scale for P1 (F = 5.64; p = 0.02), P7 (F = 8.324; p = 0.005), N1 (F = 4.95; p = 0.03), N4 (F = 4.85; p = 0.03), N5 (F = 11.17; p = 0.001), G4 (F = 6.65; p = 0.014), and the total PANSS score (F = 5.82; p = 0.018). After Bonferroni correction, the differences remained significant for P7, N5, and G4. Thus, the presence of the T allele in a homozygote or heterozygote increases the severity of the signs of P7 (1.17 ± 0.4; 3.32 ± 1.82), N5 (1.17 ± 1.03; 4.14 ± 1.79), and G4 (2 ± 1.26; 3.88 ± 1.75). In women, differences were found in the symptoms of G6—”depression” (F = 5.27; p = 0.024), but these differences did not survive Bonferroni correction.

### FAB for Rs4680 Genotypes in Youth-Onset Schizophrenia Patients

The genotype relationship was identified by five out of the six signs in the whole scale, i.e., all except the “prehension behavior (environmental autonomy)”, but after applying the Bonferroni correction, the differences remained significant only in sign 1—”similarities (conceptualization)” (F = 7.92; p = 0.006) and sign 5—”go–no go (inhibitory control)” (F = 9.64; p = 0.003). The G allele was associated with lower scores: sign 1—”similarities (conceptualization)” (2.44 ± 0.51; 1.83 ± 0.9) and sign 5—”go–no go (inhibitory control)” (2.22 ± 0.65; 1.56 ± 0.9). These features are common for women, but not for men.

## Discussion

In this study we investigated the associations with youth-onset schizophrenia of rs4680 *COMT* and rs6280 *DRD3*, which had been previously described in literature, but never before in Russians, and explorer the hypothesis that rs7322347 *5HT2A* might also be associated with schizophrenia in this population.

The link between rs7322347 and schizophrenia is described for the first time. In addition, we found that association with disease risk was significant for rs6280 and rs4680 in females and for rs7322347 in males, in case–control study. Associations of the studied polymorphisms with individual symptoms were also found. It should be noted that we used a specific population group with youth-onset schizophrenia, and therefore the associations found here may differ from those found in other cohorts.

The effect of sex steroids on the dopamine and serotonin systems has been shown in the literature (52–54). Clinical and experimental data confirm the role of steroid–dopamine interactions in the pathophysiology of schizophrenia, depression, and Parkinson’s disease (55). One study showed that oestrogens have a psychoprotective action that appears to be mediated by central dopaminergic and serotoninergic mechanisms (56). Gender-dependent differences in the incidence and symptoms of schizophrenia have been shown (57–59). Oestrogens could play a beneficial role for brain dopamine activity (55).

In our study, we found gender differences in the severity of clinical features. Higher scores for delusions (PANSS and DSM-V) and excitement (P4) were found in males and higher scores for: depression (PANSS and DSM-V), P2, G1, G5, G7, G8, and G11 were found in females. We did not find gender differences in the FAB scale. Therefore, studies of the associations of SNP of dopamine and serotonin system genes with symptoms of schizophrenia should be carried out taking into account the patient’s gender and particularly considering the sex differences found in our study. The dopamine system involvement in the development of schizophrenia has long been known (60), although it is not sufficient to account for the pathogenesis of all schizophrenia symptoms. The enzyme that carries methionine (Met/Met) retains 25% of the activity of the wild-type Val/Val enzyme. The alleles are codominant, and the enzyme activity of Val/Met heterozygotes is midway between the activities of the homozygotes (43). In our study, Val158Met polymorphism showed associations only in women, but not in the total distribution including both men and women. According to Sazci et al., (61), this polymorphism was even more significantly distributed in women with schizophrenia (p = 0.044 compared to p = 0.005 in women). The *COMT* gene encodes the COMT enzyme, which degrades catecholamines, including dopamine, especially in the prefrontal cortex (62, 63). Furthermore, it modulates dopamine levels in the frontostriatal network and, in turn, affects executive function performance and cognitive functions (64). A functional SNP rs4680, which leads to the replacement of Val158Met, may cause individual differences in emotion processing and in performance of cognitive tasks (65). In our study, the impairment of functions associated with the frontal lobe is clearly seen in the association with SNP rs4680, for all parameters in FAB scale. The presence of at least one Val, worsened cognitive impairment. After applying Bonferroni’s correction, the differences remained significant for sign 1—”similarities (conceptualization)” and sign 5—”go–no go (inhibitory control)”. Poor attention (G11) also reflects cognitive impairment; the severity of this symptom depends on the presence of the allele G in the genotype. Emotional-motivational disturbances (G8 – “uncooperativeness” and N3 – “poor rapport”) (which did not survive Bonferroni’s correction in PANSS), were also more pronounced in women with the presence of Val, which reduces enzyme activity (63). Furthermore, a link between the genotype and the total PANSS score was observed. It should be noted that these associations were found only in women. The correlation between the *COMT* genotype and psychiatric health has sex differences (65). Compared to men, women have significantly more dopamine cells within the mesocortical pathway, a major dopaminergic pathway projecting to the prefrontal cortex (50 vs 30% respectively) (66).

The *DRD3* gene is involved in the development of mental symptoms associated with the dopamine system. In our study, a weak association of SNP rs6280 with a diagnosis of schizophrenia (p = 0.03) was shown. In numerous studies, the link between rs6280 and schizophrenia (67, 68) and furthermore with youth-onset schizophrenia (69), has been described.

However, a meta-analysis conducted by Qi et al., (70) showed an absence of such correlations. In our study, the presence of the T allele (Ser/Gly and Gly/Gly) increased the severity of cognitive impairment (N5—”difficulty in abstract thinking” in PANSS) in men, by almost four times, compared to the Ser/Ser homozygotes. The DRD3 receptors is selectively localized in the limbic regions of the brain in rats, but is more widespread in humans, present in sensory, association, and motor areas, as well as limbic areas of basal ganglia. In our study, associations of rs6280 with hostility (P7 in PANSS) are also shown. The localization of DRD3 in the dopamine pathway also plays a role in the formation of emotional reactions. For example, the DRD3 receptors agonist cariprazine reduced hostility in patients with schizophrenia (71). In another study, the association between rs6280 and delusions was shown (72). The identified associations of rs6280 with G4—”tension” in PANSS in men, can be explained by the localization of the DRD3 in the nigrostriatal pathway. It should be noted that, in contrast to rs4680 *COMT*, the link between the genotype and the phenotype was detected only in men. The associations with G6 – “depression” become meaningless after correction for multiple comparisons.

Another polymorphism studied in our work is rs7322347. Until now, the available data have not shown associations with schizophrenia.

There is evidence of the possible role of rs6280 and rs7322347 interactions in the development of schizophrenia (73).

A significant link was shown with aggression in both men and women in a sample of 887 people (42). In our study, the minor allele of the *5HT2A* gene (p = 0.0001) was significantly more common among patients with early schizophrenia in the total distribution, and in men (p = 0.002). The serotonergic system has a powerful role in the pathophysiology of schizophrenia. The 5HT2A receptors is the most important excitatory receptors, regulating serotonin transmission in the brain (74). In addition, the 5HT2A also interacts with dopamine systems and indirectly increases dopamine release in the prefrontal cortex (75). The association of *5HT2A* SNP with schizophrenia may be associated with the fact that brexpiprazole, which is also an antagonist of this receptors, is effective in reducing schizophrenia relapses (76). Furthermore, antipsychotic drugs such as risperidone, olanzapine, and clozapine have high affinity for DRD2 and 5-HT2A receptors (77). Li et al., (75) showed dysregulation of *COMT and 5-HTR2A gene expression* in first-episode antipsychotic-naïve schizophrenia. Subsequent antipsychotic treatment has no significant effect on the mRNA expressions of *COMT and 5-HTR2A*. In our study, the case–control association of rs7322347 with schizophrenia was first demonstrated. Further identification of the link between the genotype and the symptoms of schizophrenia demonstrates differences in delusions in men and hostility (P7, PANSS) in women. Thus, the presence of a minor allele A significantly increases the scores for hostility but is associated with a decrease on the scale of delusions. Due to the fact that other researchers have not investigated the association of this SNP with schizophrenia, the interactions between the emotional–motivational spheres and rs7322347 have not yet been studied. Therefore, this requires further investigation.

Special features of this genetic study include the unique youth-onset schizophrenia group from the Russian population, which has not previously been investigated. The link between rs7322347 and schizophrenia was described for the first time. Gender differences in clinical features and the genetic association of rs4680 *COMT*, rs6280 *DRD3*, and rs7322347 *5HT2A* with symptoms, are of particular interest. Our assumption is that our findings hold true only for youth onset schizophrenia and that gene associations in middle and late onset schizophrenia may differ.

## Data Availability Statement

The raw data supporting the conclusions of this manuscript will be made available by the authors, without undue reservation, to any qualified researcher.

## Ethics Statement

The studies involving human participants were reviewed and approved by Interdisciplinary Ethics Committee, Moscow (22/07/2017). The patients/participants provided their written informed consent to participate in this study.

## Author Contributions

AM, KP, YZ, EZ, and OP prepared all experiments and AM and YZ wrote the manuscript. ZS: data processing. NZ, OK, AR: worked with patients. VC and GK: revised the manuscript.

## Funding

This work was supported by grant No 17-29-02164 from Russian 911 Foundation for Basic Research (RFBR).

## Conflict of Interest

The authors declare that the research was conducted in the absence of any commercial or financial relationships that could be construed as a potential conflict of interest.
